# Editorial: Epidemiological investigations of zoonotic viruses and research on drugs and vaccines

**DOI:** 10.3389/fvets.2025.1674157

**Published:** 2025-09-01

**Authors:** Jingqiang Ren, Zhenzhen Zheng, Shen Yang, Xusheng Qiu, Mingxiao Ma

**Affiliations:** ^1^Wenzhou Key Laboratory for Virology and Immunology, Institute of Virology, Wenzhou University, Wenzhou, China; ^2^Department of Medicine, Kao Autoimmunity Institute, Cedars-Sinai Medical Center, Los Angeles, CA, United States; ^3^Shanghai Veterinary Research Institute, Chinese Academy of Agricultural Sciences, Shanghai, China; ^4^College of Animal Husbandry and Veterinary Medicine, Jinzhou Medical University, Jinzhou, China

**Keywords:** zoonotic viruses, drugs, vaccines, infectious diseases, wild animals

## Introduction

Zoonotic viruses originate from wild animals and livestock. They can circulate between animal reservoir hosts and humans, leading to mild or severe illness in both species (1). In recent decades, with the emergence and re-emergence of zoonotic diseases such as Ebola, SARS-CoV, avian influenza, COVID-19, and Mpox globally (2–8), people are becoming increasingly aware of the threat these diseases pose to human health. What is astonishing is that these pathogens can be transmitted not only through direct contact but also through indirect contact or via contaminated inanimate objects. To date, no marketed drugs have been proven effective against such diseases. However, with the increasing public awareness of safety and the continuous advancement of scientific research, emergency vaccines for critical situations have been successfully developed (9). This Research Topic aims to delve into the complex mechanisms of the transmission, occurrence, mutation, and dissemination of animal pathogens within and between different populations, hoping to provide information and guidance for disease prevention and treatment.

## Vigilance against cross-species transmission of circoviruses

Circovirus is currently known as the smallest pathogenic DNA virus, traditionally referred to as porcine circovirus because it was initially isolated from pigs (10). However, circoviruses have long since breached the species barrier and have been reported in avian, aquatic, and many mammalian species, including humans (11–13). Phylogenomic and evolutionary analyses indicate that most of them are related to porcine circovirus (13). Cao et al. in this Research Topic reported a novel canine circovirus, with a code-shifting mutation occurring in the shortened ORF1 region, and a new 13-amino acid sequence emerging at the C-terminus of the replication protein (Rep), suggesting that this mutation may affect the viral replication cycle. Phylogenetic and evolutionary analyses indicate that these isolates belong to canine coronavirus genotype 3, which is more prevalent in the southeastern and southwestern regions of China as well as in neighboring countries (Cao et al.). This epidemiological investigation has broadened our understanding of the genetic diversity of the canine coronaviruses in southwestern China and provides insights into the evolution of the virus.

## Recurrent zoonotic infectious diseases

New or recurrent zoonotic infectious diseases continue to pose a serious threat to public health and the global economy, such as rabies, brucellosis, avian influenza, etc. Das et al. assessed the knowledge, attitude, and practices (KAP) of local residents regarding rabies in Turkana County, the second largest city in Kenya. The results indicated that the level of knowledge, positive attitudes, and behaviors regarding dog vaccination are all below 50% in Turkana region. The main factors influencing dog vaccination include a lack of understanding of rabies, insufficient information about immunization activities, and considerations regarding the cost of vaccination (Das et al.). These findings have significant implications for policy development and the decision-making process, emphasizing the necessity of implementing targeted interventions in such situations to enhance rabies awareness and vaccination rates.

Zakharova and Liskova employed spatiotemporal cluster analysis and a negative binomial regression algorithm to investigate the relationship between animal rabies burden and a range of environmental and socio-demographic factors across different administrative districts of Nizhny Novgorod. This approach aimed to evaluate risk factors influencing the transmission and persistence of rabies virus among wild and domestic animal populations, which is critical for developing effective strategies to control and reduce cases. The study found that the density of red foxes and the vaccination rates of wildlife and domestic animals are the most significantly associated risk factors with the severity of rabies in the study area.

## Conclusion and future perspectives

Clearly, the study and understanding of zoonotic diseases extend far beyond the topics discussed above. Although this Research Topic has only gathered a limited number of articles, it does not diminish our commitment to the critical awareness of the importance of zoonotic disease. Future research in this field requires increased investment to better elucidate the pathogenicity and molecular mechanisms of viruses, thereby promoting innovation and development, and ultimately aiding humanity in making greater progress in combating various diseases.
